# Registration of Sounds Emitted by the Madagascar Hissing Cockroach Using a Distributed Acoustic Sensor

**DOI:** 10.3390/s25072101

**Published:** 2025-03-27

**Authors:** Artem T. Turov, Yuri A. Konstantinov, Ekaterina E. Totmina, Anna G. Votinova, Grigoriy F. Masich, Dmitry A. Korobko, Andrei A. Fotiadi

**Affiliations:** 1 Perm Federal Research Center of the Ural Branch of the Russian Academy of Sciences (PFRC UB RAS), 13a Lenin St., 614000 Perm, Russia; artemtur442@gmail.com (A.T.T.); yuri.al.konstantinov@ro.ru (Y.A.K.); masich@icmm.ru (G.F.M.); 2 General Physics Department, Applied Mathematics and Mechanics Faculty, Perm National Research Polytechnic University, Prospekt Komsomolsky 29, 614990 Perm, Russia; totmina_katya@mail.ru; 3 Faculty of Biology, Perm State National Research University, Bukirev Street 15, 614990 Perm, Russia; 4 S.P. Kapitsa Research Institute of Technology, Ulyanovsk State University, 42 Leo Tolstoy Street, 432970 Ulyanovsk, Russia; korobkotam@rambler.ru; 5 Electromagnetism and Telecommunication Department, University of Mons, B-7000 Mons, Belgium

**Keywords:** fiber optic sensor, distributed acoustic sensor, DAS, insect, hissing cockroach, *Gromphadorhina portentosa*, agriculture

## Abstract

Recent advancements have expanded the applications of fiber-optic distributed acoustic sensors (DAS), including their use in monitoring the acoustic activity of insects, which can be either harmful or beneficial to agriculture. Previous studies have demonstrated the capability of DAS to record and analyze insect-generated acoustic signals in real-world conditions; however, these studies primarily involved large insect colonies. In this work, a fiber-optic DAS is used for the first time to record the sounds produced by a single insect under controlled laboratory conditions. This was achieved using an optimized and cost-effective experimental setup designed and assembled, including a specially developed and manufactured sensing element. The results demonstrate that the fiber-optic DAS effectively captures the acoustic signals of the Madagascar hissing cockroach (*Gromphadorhina portentosa*), including both the mechanical interactions of the insect with the optical fiber and the characteristic hissing sound produced in response to external stimulation.

## 1. Introduction

In the late 20th and early 21st centuries, distributed fiber-optic sensors became increasingly important in modern science and technology [1,2,3,4]. Distributed Acoustic Sensors (DAS), first introduced in 1977, have proven to be highly effective in applications such as oil and gas exploration, transportation, and processing [5,6,7], as well as in structural health monitoring [8,9,10,11,12] and perimeter security [13,14]. While DAS technology has primarily been developed for these industries, ongoing advancements have led to the emergence of new potential applications.

For instance, DAS has been reported as an indispensable tool for the early detection of red palm weevil infestations [15], outperforming traditional methods such as trained dogs, X-ray imaging, and visual inspection in terms of efficiency and scalability. Additionally, DAS technology holds great promise for monitoring individual plants on large-scale plantations [16], tracking animals and their movement patterns [17], and detecting environmental events [18,19]. A common characteristic of these emerging fields—spanning biology, ecology, agriculture, flaw detection, voice recognition, and sound engineering—is that their budgets are typically smaller than those in industries such as oil and gas, defense, and engineering. Furthermore, the sensor characteristics required for these new applications, such as acoustic frequency range, sensitivity, and sensing element length, may differ significantly from those used in traditional DAS applications. As a result, despite its unique capabilities, DAS has not yet achieved widespread adoption in biology and agriculture, even though no comparable alternative exists for certain applications.

**Figure 1:**
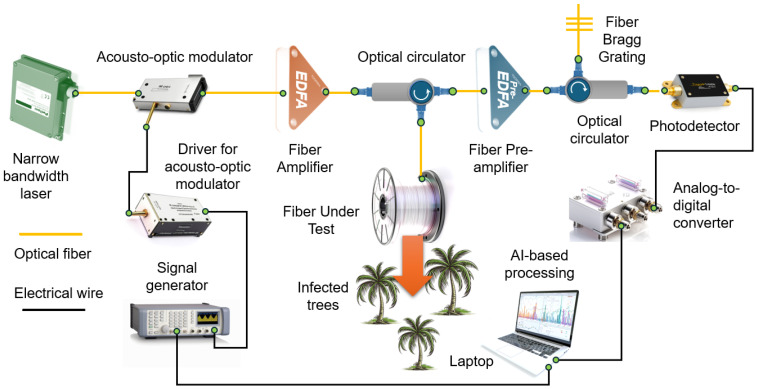
Distributed acoustic sensor (DAS) setup for the early detection of red palm weevil infestations. The system’s key components include a laser source, acousto-optic modulator, erbium-doped fiber amplifiers, optical circulators and filters, a photodetector, and a data acquisition unit. Adapted from [15].

One of the most promising real-world implementations of DAS technology is its use for the early detection of red palm weevil larvae, as demonstrated by researchers in Saudi Arabia [15]. Their setup (Figure 1) consists of a narrowband laser source, an acousto-optic modulator, erbium-doped fiber optical amplifiers, optical circulators and filters, a photodetector, a data acquisition unit, and 1 km of standard single-mode telecommunication optical fiber.

**Figure 2:**
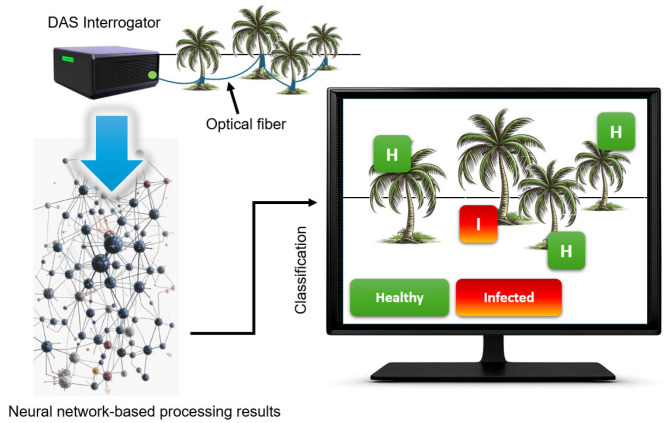
Experimental setup for the early detection of red palm weevil larvae in plantation conditions. The scheme illustrates the arrangement of optical fiber on trees and the deployment of the distributed acoustic sensor (DAS) system for monitoring acoustic emissions associated with insect activity. Adapted from [15].

Each tested tree was wound with 5 m of optical fiber (Figure 2). Prior to field deployment, the acoustic emissions of palm tree trunks infested with weevil larvae were studied under laboratory conditions using the same DAS setup. A pattern of sounds emitted by the insect was recorded at each stage of its development, allowing for the characterization of infestation-related acoustic signatures.

The signals recorded in plantation conditions were processed using a neural network, achieving an infestation detection accuracy of 97%. This demonstrates that a fiber-optic distributed acoustic sensor (DAS) is a feasible and highly effective solution for detecting tree pests at an early stage, helping to mitigate tree destruction caused by insect larvae infestations.

Traditional detection methods, such as visual inspections, pheromone traps, and spot checks, often fail to identify infestations in their early stages. Other potential early detection methods, such as X-ray imaging or trained detection dogs, are impractical for large-scale plantations, such as those of oil palms, due to scalability limitations. Acoustic monitoring has gained popularity as a technique for detecting insect activity within wood, as it enables the identification of movement and feeding sounds. Research efforts have explored the use of contact microphones and vibration sensors for this purpose. However, deploying traditional microphones to monitor each tree individually would be economically unfeasible compared to a fiber-optic sensor.

Fiber-optic sensors and distributed acoustic sensing systems based on optical fibers are becoming increasingly popular in various fields, including seismic monitoring, infrastructure diagnostics, and security. One key advantage of DAS technology is its ability to monitor entire plantations or farms using a single sensor or a small number of sensing elements. However, the application of DAS for detecting insect activity in forest ecosystems remains a relatively new and promising research area. This is due to the fact that the sensor requirements in biology and agriculture differ significantly from those used in fields such as mineral extraction and perimeter security.

Currently, no reports have been found on the detection of individual insects using fiber-optic DAS through airborne acoustic signals or direct contact between the insect and the sensing element. Previous studies, such as the one described above, have focused on detecting infestations by groups of larvae through their acoustic emissions within the wood. Therefore, the objective of this study is to investigate the feasibility of registering the acoustic signature of a single insect using a fiber-optic DAS of a “classical” design outside of a woody environment.

This research will help evaluate the accuracy of the experimental setup and its parameters, as well as assess the potential of fiber-optic DAS for detecting insects through their acoustic emissions, not only in large colonies within sound-conducting environments but also in more diverse and less conventional settings.

The Madagascar hissing cockroach (*Gromphadorhina portentosa*) was selected as the subject of this study for several reasons:

- Large size. The Madagascar hissing cockroach is one of the largest cockroach species in the world, with some individuals reaching up to 10 cm in length;
- Ability to produce hissing sounds. This species uses hissing as its primary defense mechanism against insectivorous predators;
- Lack of wings. Unlike many other cockroach species, Madagascar hissing cockroaches have lost the ability to fly, as wings are unnecessary for burrowing in forest litter. Instead, they have developed a thick and durable chitinous exoskeleton;
- Suitability for experiments. Due to their large size and wingless nature, Madagascar hissing cockroaches have been widely used in scientific research. For instance, Japanese scientists selected this species for experiments in developing remote-controlled cyborg cockroaches, as they are large enough to carry equipment without the interference of wings [20].

## 2. Materials and Methods

### 2.1. The Madagascar Hissing Cockroach

**Figure 3:**
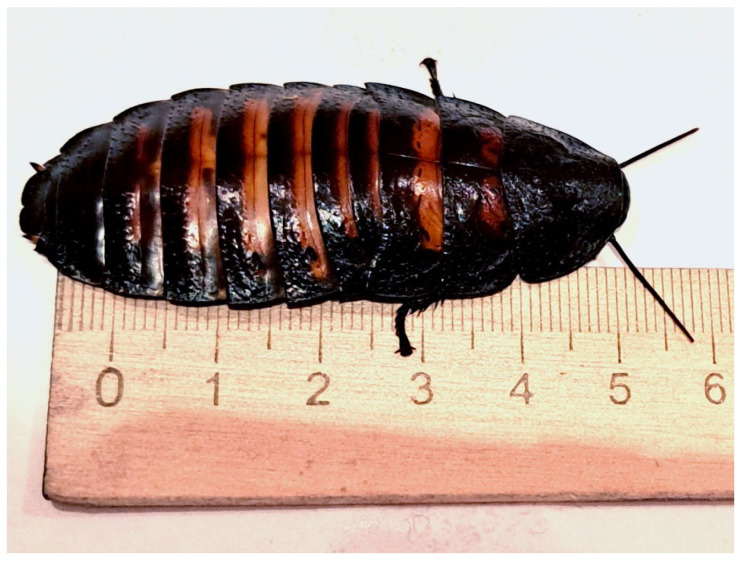
A male *Gromphadorhina portentosa* (Madagascar hissing cockroach) was used in the study. The insect’s acoustic emissions and movements were recorded using a fiber-optic distributed acoustic sensor (DAS).

The Madagascar hissing cockroach (*Gromphadorhina portentosa*) (Figure 3) is a large tropical insect that lives on the trunks and branches of trees and bushes and feeds on green shoots and fruits. Individuals of this species move slowly and have no wings. When sensing danger, the cockroach freezes and makes a loud hiss. The sound is produced by a sharp contraction of the abdomen, forcing air through the spiracles. Sound signals are also used for intraspecific communication (for example, the fight between males for females) [21]. This cockroach is one of the largest representatives of the cockroach family in the world. Its body length is, on average, about 4–5 cm, but can reach 10 cm. The insect weighs up to 40 g.

**Figure 4:**
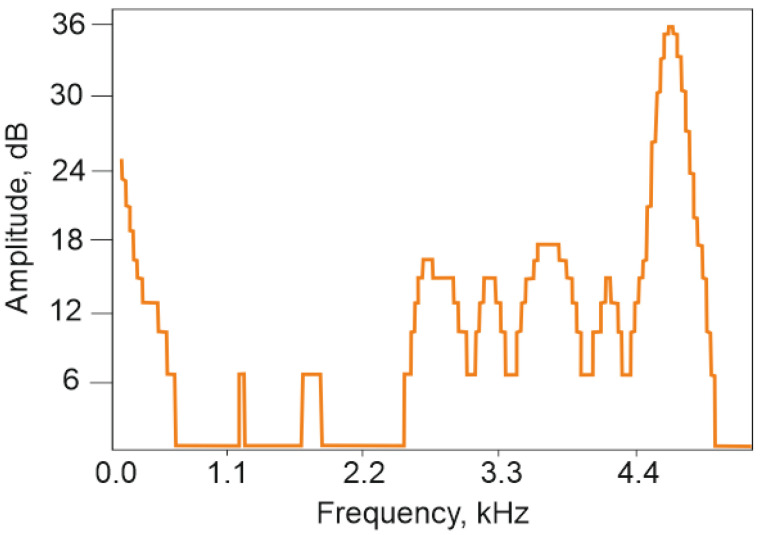
Amplitude-frequency dependence of *Gromphadorhina portentosa* hiss at a specific point in time. The spectrum illustrates the frequency components of the insect’s acoustic emission. Adapted from [22].

The acoustic signature of the cockroach’s hiss spans a broad frequency range (0.8–4.6 kHz), characterized by blurred edges, narrow peaks concentrated in the center, and compactly arranged triangular expansions. In some cases, two or three additional peaks appear, particularly along the high-frequency edge (Figure 4) [22].

The spectrum of the cockroach hiss slightly changes within its duration. Moreover, it depends on the size, age, and other features of a cockroach individual. Nevertheless, the spectrum of *Gromphadorhina portentosa* hiss is quite repeatable, especially in terms of main harmonics such as 4.9 kHz (Figure 4).

### 2.2. Distributed Acoustic Sensing

To better understand the principles underlying the distributed acoustic sensor used in this study, this section presents the theoretical foundation of phase-sensitive optical time-domain reflectometry (Φ-OTDR). Distributed phase-sensitive optical time-domain reflectometry utilizes coherent light backscattered from an optical fiber to detect and analyze perturbations induced by external physical fields. This technique enables real-time, distributed acoustic sensing by continuously monitoring changes in the backscattered signal caused by external influences along the fiber length.

The Φ-OTDR technique enables distributed acoustic sensing by utilizing coherent Rayleigh backscattering from an optical fiber. This method is employed for detecting and analyzing perturbations induced by external physical fields along the fiber length. It involves injecting probe pulses from a highly coherent laser source into the sensing fiber, where each pulse interacts with inhomogeneities in the fiber’s refractive index. These interactions result in a scattered signal that is recorded at the fiber input and analyzed to localize and quantify external forces acting on the fiber.

In a Φ-OTDR system similar to that used in the experiments, a probe pulse is injected into an optical fiber that exhibits low linear losses $\alpha \left(x\right)$ distributed along its length. At the initial moment t=0, the leading edge of the pulse is positioned at the fiber input x=0. The probe pulse is assumed to have a rectangular shape, with $I_{1}$ the peak pulse intensity and T the pulse duration. As the pulse propagates through the fiber, it undergoes Rayleigh backscattering due to multiple reflections from microscopic refractive index variations that were frozen into the fiber during manufacturing. These variations can be modeled as Rayleigh reflection centers with a relative reflectivity $\sim \rho \left(x\right),$ randomly distributed along the fiber length.

At a given time $t_{0}+T,$ the leading edge of the pulse reaches the fiber position

(1) $$x_{1}=\frac{c}{n}\left(t_{0}+T\right),$$

while the trailing edge is at

(2) $$x_{0}=\frac{c}{n}t_{0}.$$

The light backscattered by the distributed reflection centers creates an interference pattern within the pulse. The backscattered intensity measured at the pulse’s trailing edge is denoted as $I_{2}\left(t_{0},x_{0}\right).$ The complex amplitude $E_{2}\left(t_{0}+T,x_{0}\right)$ of the electric field associated with $I_{2}\left(t_{0}+T,x_{0}\right)$ is given by the superposition of all backscattered fields generated by the pulse at earlier times and arriving at $x_{0}$ precisely at $t_{0}+T$. The backscattering events that contribute to this superposition occur over a time interval between $t_{0}+\frac{T}{2}$ and $t_{0}+T.$ Thus, the total backscattered electric field is

(3) $$E_{2}\left(t_{0}+T,x_{0}\right)=\int_{0}^{\frac{T}{2}}E_{1}\left(t_{0}+T-\tau ,x_{0}+\frac{c}{n}\tau \right)\rho \left(x_{0}+\frac{c}{n}\tau \right)d\tau .$$

Here $E_{1}\left(t,x\right)$ represents the complex amplitude of the probe pulse, modeled as a segment of a monochromatic optical wave at frequency

(4) $$\omega_{0}=2\pi f_{0}=\frac{2\pi }{\lambda_{0}}\frac{c}{n},$$

where $\lambda_{0}$ is the laser wavelength. The pulse propagates through the single-mode optical fiber with a group velocity of $\frac{c}{n},$ following

(5) $$E_{1}\left(t,x\right)=\sqrt{\gamma \left(x\right)I_{1}}\exp\left\{i\omega_{0}\left(t-\frac{n}{c}x\right)\right\}\mathrm{for}\;t\in \left[\frac{n}{c}x;\;\frac{n}{c}x+T\right],\;x\in \left[0,\;L\right],$$

where the integrated loss factor is expressed as

(6) $$\gamma \left(x\right)=\exp\left(-\int_{0}^{x}\alpha \left(x^{\prime }\right)dx^{\prime }\right).$$

Substituting this into the equation for $E_{2}\left(t_{0}+T,x_{0}\right)$ results in the expressions for the backscattered signal amplitude and intensity at the probe pulse trailing edge:

(7) $$E_{2}\left(t_{0}+T,x_{0}\right)=\sqrt{\gamma \left(x_{0}\right)I_{1}}\exp\left\{i\left(\omega_{0}\left(t_{0}+T-\frac{n}{c}x_{0}\right)\right)\right\}\int_{0}^{\frac{T}{2}}\exp\left\{-i2\omega_{0}\tau \right\}\rho \left(x_{0}+\frac{c}{n}\tau \right)d\tau ,$$

(8) $$I_{2}\left(t_{0}+T,x_{0}\right)=\gamma \left(x_{0}\right)I_{1}\int_{0}^{\frac{T}{2}}\int_{0}^{\frac{T}{2}}\exp\left\{-i2\omega_{0}\left(\tau -\tau^{\prime }\right)\right\}\rho \left(x_{0}+\frac{c}{n}\tau \right)\rho^{*}\left(x_{0}+\frac{c}{n}\tau^{\prime }\right)d\tau \;d\tau^{\prime }.$$

Once formed, these amplitude and intensity quantities propagate to the fiber input without modification of the interference structure just exhibiting linear losses.

The detected intensity $I_{D}\left(t_{D}\right),$ recorded at the fiber input at $t_{D}=\left(2t_{0}+T\right),$ depends on the Rayleigh backscattering from a fiber segment $\left[x_{D}-\frac{\Delta }{2},x_{D}+\frac{\Delta }{2}\right],$ where

(9) $$x_{D}=\frac{c}{2n}\left(t_{D}-\frac{T}{2}\right)$$

is the mean interval point, and the probe pulse duration T sets the spatial resolution

(10) $$\Delta =\frac{c}{n}\frac{T}{2}$$

of the described sensing technique.

The intensity detected at the fiber input at the moment $t_{D}$ can be expressed as

(11) IDtD=2I1γ2xD∫ΛT2Reexp−i2ω0ΔτFΔτ, xDdΔτ+I1ΛF0, xD,

where the first and second terms describe the coherent and incoherent parts of the Rayleigh scattering process.

The function

(12) $$F\left(\Delta \tau ,\;x_{D}\right)=\int_{-\frac{T}{4}+\frac{\Delta \tau }{2}}^{\frac{T}{4}-\frac{\Delta \tau }{2}}\rho \left(x_{D}+\frac{c}{n}\left(\tau +\frac{\Delta \tau }{2}\right)\right)\rho^{*}\left(x_{D}+\frac{c}{n}\left(\tau -\frac{\Delta \tau }{2}\right)\right)\;d\tau$$

is the autocorrelation function that characterizes the distribution of Rayleigh backscattering centers in an undisturbed fiber. Importantly, for any given fiber, this function is specific and is commonly considered the “fingerprint” of the Rayleigh sensor.

One can see that the coherent part of the detected intensity signal $I_{D}\left(t_{D}\right)$ is very sensitive to fluctuations of the probe laser frequency $\omega_{0}$ and to changes in mutual positions of the reflection centers $∼\rho \left(x\right).$ This inherent sensitivity underlies the use of the Φ-OTDR for distributed sensing. The external perturbations affect the mutual positions of the reflection centers $∼\rho \left(x\right)$ that, in turn, affect the signal $I_{D}\left(t_{D}\right).$ To measure these perturbations, the fiber is interrogated by periodic coherent probe pulses with the period W. When sensing fiber is perturbated, the distribution of the reflection centers $∼\rho \left(x\right)$ slightly varies. Such perturbation does not affect the incoherent part of the detected signal but does affect its coherent part. Mathematically, it can be expressed as a substitution of

(13) $$\Delta \tau \to \left(1+s\left(x_{D}\right)\right)\Delta \tau$$

into the function $F\left(\Delta \tau ,\;x_{D}\right),$ where $s\left(x_{D}\right)<<1$ ($\sim 10^{-6}$) is a small variable parameter evaluating the effect of the external force on the fiber at point $x_{D}.$ As the external force varies in time, it modulates $s\left(x_{D},t\right).$ Therefore, the signals $I_{D}\left(t_{D,m}\right)$ detected from different consequent probe pulses express the modulation

(14) $$\delta I_{D}\left(t_{D,m}\right)\sim s\left(x_{D,}t_{D,m}\right),$$

where m=0, 1, 2... is the probe pulse number. The sensitivity is expressed as

(15) KxD=dIDdstD=−dIDdω0tDω0=−4I1γ2xDω0∫ΛT2ΔτImexp−i2ω0ΔτFΔτ, xDdΔτ.

Although the sensitivity $K\left(x_{D}\right)$ of the signal modulation $\delta I_{D}\left(t_{D,m}\right)$ to the modulation of $s\left(x_{D}t_{D,m}\right)$ depends on the fiber point $x_{D}$ one can see that when an external force modulates $s\left(x_{D}t_{D,m}\right)$ periodically, the recorded signal $I_{D}\left(t_{D,m}\right)$ also exhibits periodic modulation. This mechanism enables the extraction of acoustic spectra applied to the fiber by analyzing the modulation patterns $I_{D}\left(t_{D,m}\right).$ It is clear that to this effect, the external field modulation frequency should be much lower than the repetition rate

(16) $$R=\frac{1}{W}$$

of the pulse interrogation. In this case processing of the signal $I_{D}\left(t_{D,m}\right)$ for different points $x_{D}$ along the fiber enables distributed reconstruction of the acoustic spectra applied to the sensing fiber.

Thus, the described mechanism explains the ability of Φ-OTDR to detect weak acoustic signals over long distances, making it a promising tool for distributed acoustic sensing applications. Given its capability to detect weak acoustic signals across long distances, Φ-OTDR is an ideal technique for applications such as environmental monitoring, structural health assessment, and biological studies. In the present work, it has been applied to analyze the acoustic emissions of a single Madagascar hissing cockroach, which underscores the system’s high sensitivity and resolution. This demonstrates the feasibility of distributed fiber-optic acoustic sensors in bioacoustics research, extending their applications beyond conventional industrial and security domains.

### 2.3. Experimental Setup

A laboratory-based fiber-optic distributed acoustic sensor (DAS) was used for the experiments. Its operating principle is based on Φ-OTDR with direct detection (without phase extraction). This design enables the recording of acoustic signals within the target frequency range (200–5000 Hz) with acceptable quality. Additionally, the use of a pulsed current-driven laser, along with the absence of an optical modulator and fiber Bragg gratings, reduces both setup costs and sensitivity to environmental disturbances. The implementation of a low-cost system design [23,24,25] is particularly important for expanding the use of distributed fiber-optic sensors in biology and agriculture.

The system utilized a GSPF 053 arbitrary waveform generator (Rudnev-Shilyaev, LLC, Moscow, Russia) to generate current pulses with a duration of 40 ns and a repetition period of 11 μs. These pulses were fed into a pulsed laser source (DL-BF12-CLS101B-S1550, DenseLight Semiconductors, Singapore), which had an output power of 10 mW, a wavelength of 1550 nm, and a bandwidth of 5 kHz. The laser converted the electrical pulses into optical pulses, which were subsequently amplified to 30.5 mW by an erbium-doped fiber amplifier (AEDFA-23-M-FA, Amonics Ltd., Hong Kong, China). The amplified pulses were then injected into the sensing element—Corning SMF-28 optical fiber—through an optical circulator (Advanced Fiber Resources, Ltd., Zhuhai, China).

**Figure 5:**
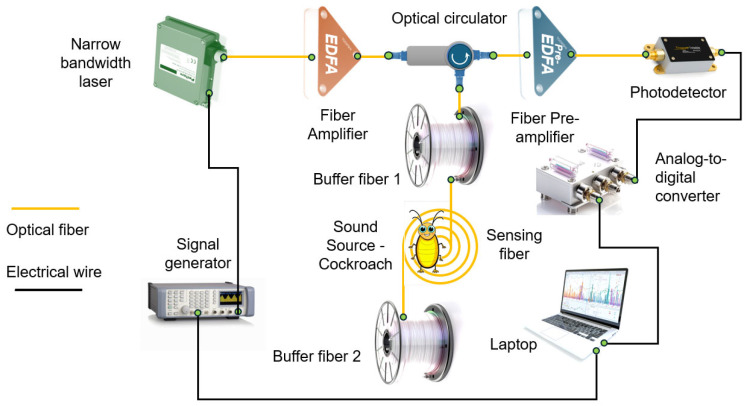
The distributed acoustic sensor (DAS) setup used in the experiment. In contrast to setup from [15], it does not require the use of acousto-optic modulator, its driver, and a fiber Bragg grating filter.

Within the fiber, due to inherent refractive index inhomogeneities [26], light pulses underwent Rayleigh scattering. Given the narrow laser bandwidth, the scattered light components within each pulse length interfered with one another [27]. The time-domain amplitude of the resulting backscattered signal depended on the distribution, quantity, and size of inhomogeneities along the fiber. A portion of this backscattered light traveled back to the fiber’s input end, where it was redirected via an optical circulator to an Amonics AEDFA-35-M-PA erbium-doped fiber preamplifier (Amonics Ltd., Hong Kong, China). The preamplified backscattered signal, reaching a power of 1.99 mW, was detected by a Thorlabs PDA 10D-EC photodetector (Thorlabs Inc., Newton, NJ, USA) and then digitized using a La-n1usb analog-to-digital converter (ADC) (Rudnev-Shilyaev LLC, Moscow, Russia) before being processed on a personal computer (Figure 5).

**Figure 6:**
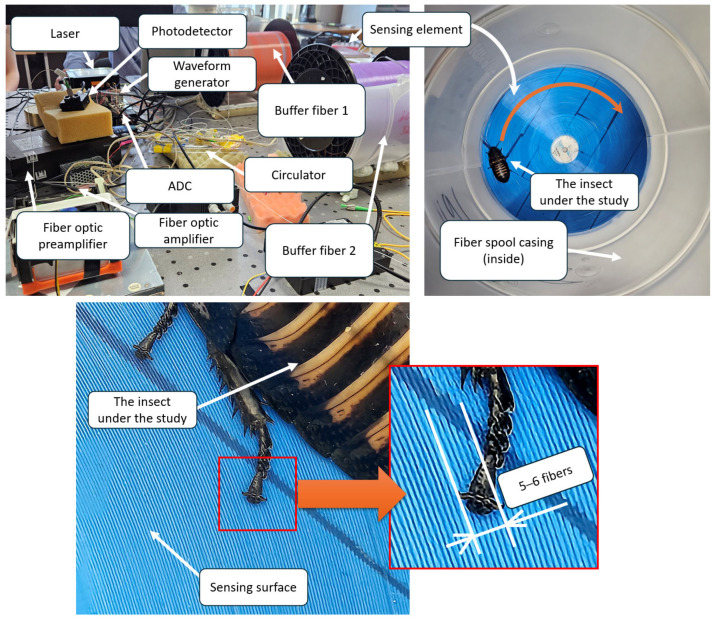
Photograph of the experimental setup, showing the sensing element and the *Gromphadorhina portentosa* (Madagascar hissing cockroach) used in the study. The fiber-optic sensor was configured to detect and analyze the insect’s acoustic emissions and movements.

The ADC operated at a sampling frequency of 500 MHz, corresponding to a spatial resolution of approximately 0.2 m, and featured a buffer capacity that allowed continuous acquisition of up to 762 traces. The unprocessed output signal represented the dependence of scattering intensity on time for each point along the optical fiber. When an acoustic signal was present, it modulated both the position and magnitude of refractive index inhomogeneities within the optical fiber at that location, with the modulation occurring at the same frequency as the acoustic signal itself. Consequently, the Rayleigh backscattered signal from this location was also modulated at the same frequency. By utilizing knowledge of the speed of light in the given optical fiber and applying a fast Fourier transform (FFT), it was possible to determine the location, amplitude, and frequency of the acoustic signal affecting the fiber. The design of the sensing element (Figure 6) plays a crucial role in optimizing signal detection and requires a detailed description.

For high-quality signal detection using a distributed acoustic sensor (DAS), the optical fiber must be in direct physical contact with the insect under study. Since the objective was not only to record the acoustic signals emitted by the insect but also to localize its position within a defined area, it was essential to ensure that physical contact between the insect and the fiber-optic sensor could occur at any point within the sensing region. This was achieved by using a standard single-mode optical fiber with an acrylate coating, and an outer diameter of 250 μm was arranged in a spiral configuration on a flat surface. Thus, the fiber used was bare (not cabled), and the expected sensitivity of the system was relatively high. The method for forming the fiber-optic spiral was as follows. A 30 cm diameter vinyl record was used as the sensing platform. This choice allowed the setup to be installed on a music player flywheel, enabling controlled rotation of the record if needed. To securely attach the optical fiber to the disk, a double-sided adhesive tape with a polypropylene base (50 mm × 10 m) was used. This tape featured an adhesive layer on both sides, providing a strong yet manageable connection between the vinyl record and the optical fiber. The attachment process involved fully covering the surface of the vinyl record with one side of the adhesive tape. The protective layer on the exposed adhesive side was then gradually removed as the optical fiber was affixed in concentric circles, starting from the center of the record and moving outward toward the perimeter. The most labor-intensive step was ensuring a secure and precise placement of the fiber at the contact points with both the adhesive tape and the previously adhered fiber loops. A rod tool with a smooth convex end was used to press the fiber against the adhesive at a 45-degree angle to the record’s surface to achieve this. This approach allowed one hand to fix the fiber’s contact point while the other hand rotated the record, ensuring an even and tightly packed spiral pattern. The individual fiber loops were positioned as closely as possible to maximize the sensing area. Approximately 200 m of optical fiber were successfully placed onto the vinyl record using this method, forming a compact and efficient sensing element.

**Figure 7:**
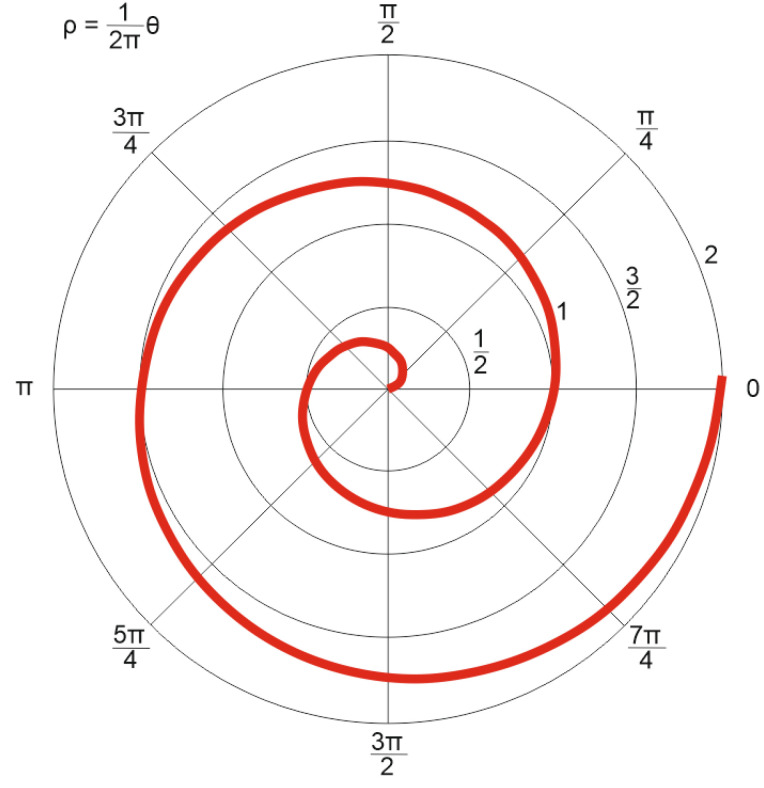
Archimedean spiral in the polar coordinate system. The spiral represents the layout of the fiber-optic sensing element used in the experiment, where each successive turn is equidistant from the previous one, enabling accurate localization of acoustic events.

The sensing element was fusion-spliced at both ends to two buffer sections of single-mode fiber wound on transport spools, each approximately 400 m long. This was carried out to prevent dead zones in the DAS system, which are caused by strong signal reflections at both the input and output, from interfering with the studied area. Additionally, to minimize light reflections at the fiber output, the fiber end was immersed in a container filled with glycerin, effectively reducing unwanted optical feedback. The sensor’s perimeter was enclosed within the casing of the optical fiber transport spool to restrict the insect’s movement during the experiment. This ensured that the insect remained within the designated sensing area, allowing for accurate measurements. For precise localization of the insect relative to the sensing element, proper data processing was required. The dense spiral arrangement of the optical fiber on the vinyl record, as described earlier, follows the geometry of an Archimedean spiral (Figure 7). A key property of this spiral is that any radial line drawn from the center intersects successive turns of the spiral at equidistant points, with this spacing referred to as the spiral pitch. This property plays a crucial role in interpreting the insect’s position within the sensing area.

The equation of an Archimedean spiral in the polar coordinate system is given by:

(17) $$\rho =\frac{a}{2\pi }\varphi ,$$

where ρ is the radius, φ is the angle in radians, a is the spiral path, representing the radial displacement per full revolution. Since the developed data processing program outputs the image as colored pixels, it is more convenient to express Equation (17) in parametric form:

(18) $$\left\{\begin{array}{c} x=\rho \cos(\varphi )\\ y=\rho \sin(\varphi ) \end{array}\right\},$$

where *x* and *y* represent the pixel coordinates in the Cartesian plane. When constructing the sensing element model, the initial and final values of the spiral radius and its pitch are defined in the program. The spiral is generated by incrementing the angle φ in small steps (0.01 rad), causing a gradual increase in the radius ρ until it reaches its final value. Simultaneously, the arc length *l* of the optical fiber laid in the spiral accumulates as:

(19) $$d\rho =\frac{a}{2\pi }d\varphi .$$

(20) $$dl=\frac{a}{2\pi }\sqrt{1+\varphi^{2}}d\varphi .$$

Each arc segment of the spiral is assigned an RGB color value corresponding to the normalized amplitude of the acoustic signal recorded at that specific location. The color of the *i*-th section of the spiral is expressed as:

(21) $$\left\{255A\left[i\right];\;0;\;255\left(1-A\left[i\right]\right)\right\},$$

where *A* represents a set of normalized signal amplitude values recorded by the fiber sensor. Thus, when visualizing the data, the program colors sections of the fiber spiral where the acoustic signal amplitude is maximum in red, while sections with minimal amplitude are colored blue.

## 3. Results and Discussion

**Figure 8:**
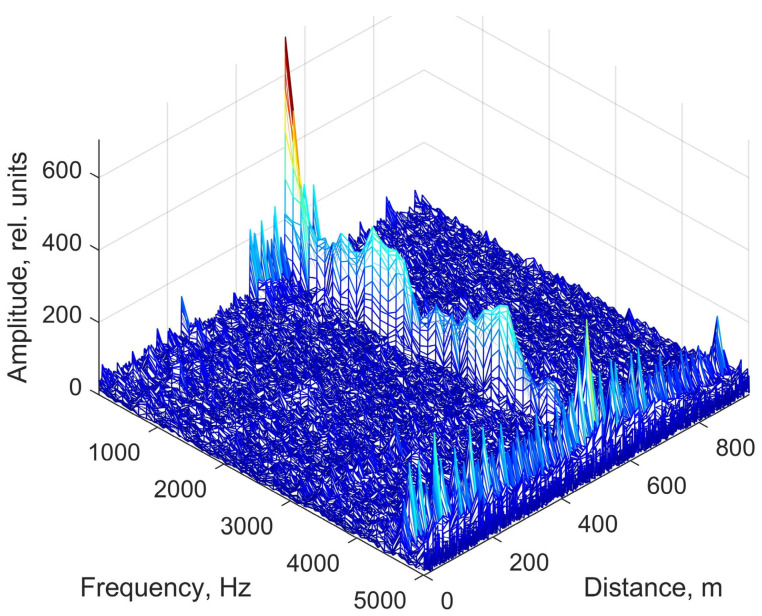
Hissing and rapid movement of a *Gromphadorhina portentosa* (Madagascar hissing cockroach) recorded using the distributed acoustic sensor (DAS). The figure illustrates the acoustic signals generated by the insect’s hissing and physical interaction with the fiber-optic sensing element.

The insect under study, a male Madagascar hissing cockroach measuring 65 mm in length and weighing 27 g, was placed onto the sensor element. Initially, the cockroach exhibited minimal movement; however, after an adaptation period of approximately 10 min, it began to move slowly across the sensor surface. The observed movement trajectory was primarily concentrated near the outer radius of the plate. This behavior suggests that in an unfamiliar environment with an unstable surface for movement, the insect was drawn toward potential escape routes or attempted to use the inner surface of the spool casing as a shelter. The fiber trace was visualized in real time on the system display. It is important to note that slow movements (approximately up to 1 cm/s) were barely visible on the spatial scan, likely due to the lower amplitude of the resulting acoustic signal. To analyze the cockroach’s response to external stimuli, hissing and rapid movements were induced multiple times during the experiment. The resulting signal, recorded and processed according to the previously described methodology, is presented in Figure 8.

**Figure 9:**
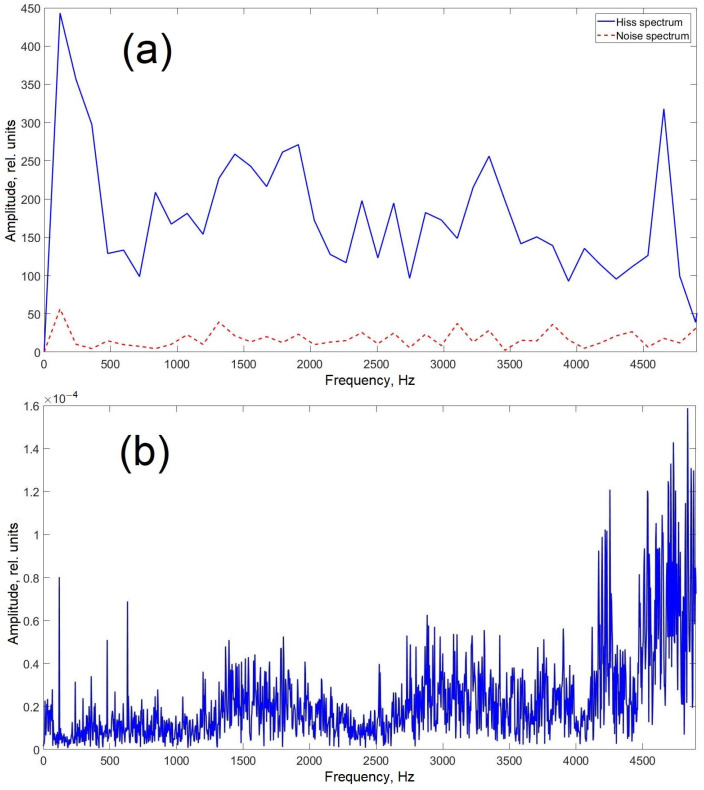
(**a**) Frequency spectra of the hiss and external noise recorded at a distance of 542.2 m along the fiber, showing the acoustic response detected by the distributed acoustic sensor (DAS); (**b**) spectrogram of the *Gromphadorhina portentosa* hiss recorded using a smartphone, serving as a reference for comparison.

The output data from distributed acoustic sensing (DAS) systems are most effectively represented in frequency-distance-amplitude coordinates. This visualization method allows a single image to encapsulate all key characteristics of the acoustic signals detected by the sensor. In the low-frequency region, it is evident that the entire sensing element exhibits an elevated noise background compared to the buffer spools. This background noise is independent of the presence of the insect on the sensing element. The most likely explanation for this phenomenon is that the current sensor design functions as a highly responsive membrane, efficiently transmitting vibrations from the optical table, including minor oscillations from auxiliary equipment. Despite this, the background noise did not interfere with obtaining the desired results. On the contrary, its localized presence in the low-frequency region allows for a preliminary estimation of the insect’s position relative to the entire sensing element. Figure 8 confirms that the insect remained closer to the outer edge of the sensing element. This conclusion is supported by the fact that the first buffer spool was fusion-spliced to the center of the spiral-laid fiber, while the second spool was connected to its outermost part. Additionally, the recorded data show interference propagating along the entire fiber length, including the buffer spools, at approximately 5000 Hz. This interference is likely of electrical origin and will not be considered in further analysis. Importantly, its presence does not obstruct the detection of the insect’s acoustic signature, as the useful signal level remains higher than the average noise level in the affected region. Figure 9a shows the acoustic spectrum of the studied insect hiss signal inherent in the spatial cross-section at a distance of 542.2 m, as well as the spectrum of external noise recorded at this location in the absence of an insect. Notably, the signal shape in this frequency range closely resembles previously reported data (see Figure 4). The dominant frequency peaks observed at 100, 1500, 3300, and 4500 Hz align well with the spectrogram of the insect’s hiss, which was independently recorded using a smartphone (SM- A536E/DS, Samsung Electronics Co., Ltd., Suwon, Republic of Korea) during the experiment (Figure 9b).

**Figure 10:**
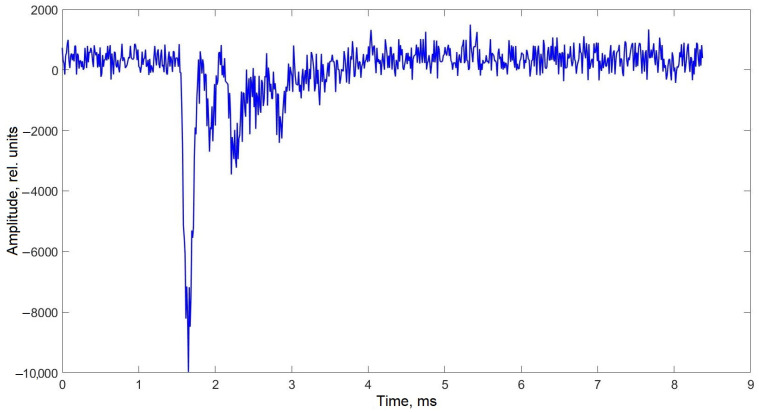
Time-domain representation of the signal recorded at 542.2 m along the fiber (corresponding frequency-domain representation can be found in Figure 9a). The plot illustrates the temporal characteristics of the acoustic signal detected by the distributed acoustic sensor (DAS), corresponding to the hissing and movement of *Gromphadorhina portentosa*.

The frequency spectrum recorded by the fiber-optic sensor contains a limited number of data points. This is due to the relatively small number of available time-domain samples, which are recorded sequentially and continuously. The primary constraint stems from the limited buffer capacity of the ADC, making it a hardware limitation inherent to the current system design. This limitation could be mitigated by using an ADC with a larger buffer and improved specifications. However, it is important to consider that upgrading the ADC would increase the overall cost of the setup. Despite this constraint, the system remains capable of recording and distinguishing the most characteristic frequencies of the acoustic signals generated by the insect (Figure 9). Additionally, the time-domain representation of the insect’s acoustic signal, presented in Figure 10, may provide further insights into the temporal dynamics of its sound production. Considering it, together with the spectrum analysis of this signal and background noise, one can distinguish between the frequency components generated by the insect and those resulting from environmental or equipment-related noise.

**Figure 11:**
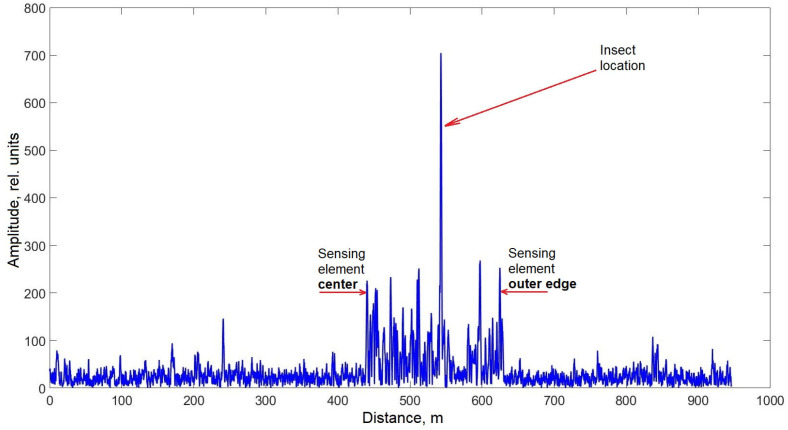
Frequency-domain cross-section of the data from Figure 8 at 119.3 Hz. Red arrows with captions indicate the location of the sensing element along the setup fiber optic part and studied insect on it.

The dip at the beginning of the signal in Figure 10, as well as the low-frequency peak around 100 Hz observed in Figure 8 and Figure 9, is likely not directly related to the hissing sound produced by the cockroach. Instead, it is presumably associated with the insect’s rapid movement, which typically coincides with hissing. This suggests that the recorded signal includes components resulting from the physical contact of the insect’s legs with the optical fiber rather than purely airborne acoustic emissions. As previously mentioned, the elevated low-frequency noise level detected by the sensing element facilitated signal localization within the sensor’s output data (Figure 11) and provided an approximate estimation of the cockroach’s position (Figure 8).

**Figure 12:**
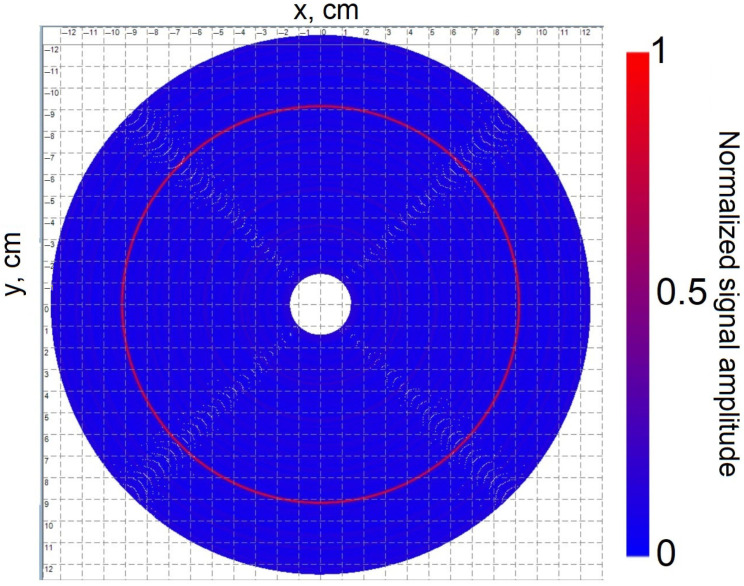
Localization of the *Gromphadorhina portentosa* (Madagascar hissing cockroach) acoustic signal relative to the sensing element. The distributed acoustic sensor (DAS) detected and mapped the insect’s hissing and movement, demonstrating the system’s capability to determine the sound source position.

The sensing element exhibited higher noise sensitivity compared to the buffered fiber, which not only facilitated precise detection of acoustic signals but also allowed for an accurate determination of the fiber length laid in a spiral on the vinyl record: 192.25 m. This information was crucial for calibrating the program responsible for localizing the sound source along the spiral. The output data from the localization program, which maps the detected effect relative to the sensing element, are presented in Figure 12.

The fiber transport spool casing, which restricted the insect’s movement, had a diameter of approximately 23 cm. Figure 12 confirms that the system successfully detected and localized the cockroach’s movements and sounds near the casing. However, the localization accuracy was inherently limited to the diameter of the circular region within which the impact was registered. The circumference of this circular region is approximately 0.6 m, which is significantly shorter than the 8 m light pulse length generated by the laser. This suggests that, in this case, the accuracy of impact localization is primarily limited by the ADC sampling rate rather than the pulse length. As previously noted, the ADC sampling rate used in this setup results in a spatial resolution of 20 cm. Consequently, the acoustic signal produced by the insect was captured in only three ADC samples, limiting the precision of its localization. The cockroach was not harmed during the experiments. After the study, it was returned to an optimal environment.

Considering the length of the fiber segment within which the cockroach’s hissing was detected (0.6 m) and assuming the insect acts as a pointwise acoustic source, one can conclude that the system’s localization precision is roughly 0.3 m along the fiber length. This value is in reasonable agreement with the system’s spatial resolution of 0.2 m. Localization precision in polar coordinates can also be considered. Thus, the radial localization precision is significantly high—comparable to the diameter of a single spiral layer (approximately 250–300 μm)—while the angular localization error is relatively large, up to 180 degrees, due to the symmetric nature of the spiral layout.

## 4. Conclusions

Despite the aforementioned limitations, primarily related to the ADC characteristics and signal noise, the fiber-optic sensor and the proposed sensing element design successfully demonstrated the ability to record the acoustic signature of a single insect. Future research will focus on determining which parameter contributes more significantly to the inaccuracy in localizing the sound source and estimating the impact length on the fiber. The current laser source is incapable of producing light pulses shorter than 20 ns, while the ADC sampling rate could potentially be increased to 2 GHz. However, this would significantly reduce the volume of continuously recorded data, a limitation that could be mitigated by reducing the length of the optical fiber connected to the sensor. The successful recording of an acoustic signal from a single insect marks an important step in the development of new applications for fiber-optic acoustic sensors. Previous studies have either recorded entire insect colonies or individual subjects of considerably larger size and weight, which inherently emit higher-intensity acoustic vibrations. To extend this technique to even smaller organisms than the Madagascar hissing cockroach, enhancements in system sensitivity and signal processing will be necessary. International research efforts suggest that neural networks may play a key role in optimizing signal detection and analysis. Additionally, an important hardware modification would be to implement a hybrid detection scheme, enabling the extraction of optical phase information instead of merely measuring signal intensity.

The approach tested in this study, utilizing the Madagascar hissing cockroach, serves as a foundation for future research and the development of methods for species identification and invasive species detection. While previous studies have monitored insects acoustically, they have relied on conventional hardware that records sound at a single point where the insect is localized or fixed. In contrast, the fiber-optic sensor in this study not only records sound waves emitted by the insect but also localizes them on a plane. This work paves the way for distributed monitoring of insect habitats created by humans. Beyond agricultural plantations vulnerable to pest infestations [28], potential applications include beehive monitoring. The global decline in bee populations is an increasingly urgent issue, and recent studies have begun investigating bee behavior through acoustic analysis [29]. The approach demonstrated in this study could significantly enhance the functionality of such research, contributing to the broader field of bioacoustics and ecological monitoring.

Future research will also focus on signal processing and impact classification. Presumably, two main challenges must be addressed to achieve this goal. The first is the accurate classification of interference caused by equipment operation or other background sources. This interference can be identified by analyzing signal spectra using Fourier and wavelet transforms, as well as by decomposing the signal into components using empirical mode decomposition (EMD) and variational mode decomposition (VMD). To enhance the signal-to-noise ratio, dynamic filtering techniques such as Frequency Domain Dynamic Averaging (FDDA) and Activation Function Dynamic Averaging (AFDA), developed and tested by our research group, will be applied. Once a satisfactory signal-to-noise ratio is achieved, neural network-based recognition methods will be employed. As demonstrated in studies on the red palm weevil, neural networks can be trained in controlled laboratory conditions and later applied in the field to detect and classify acoustic signatures of specific biological targets in the presence of diverse ambient noise.

## Data Availability

The data reported in this manuscript are available on request from the corresponding author.
